# Supplementary material for: Sex Matters–Insights from Testing Drug Efficacy in an Animal Model of Pancreatic Cancer
Source: Cancers (Basel). 2024 May 16;16(10):1901. doi: 10.3390/cancers16101901 (PMC11120498; doi:10.3390/cancers16101901)
Supplement: Supplementary file 1 [file cancers-16-01901-s001.zip › cancers-3009602-supplementary.pdf]

cancers

MDPI

Supplementary Materials

Sex Matters–Insights from Testing Drug Efficacy in an Animal Model of Pancreatic Cancer

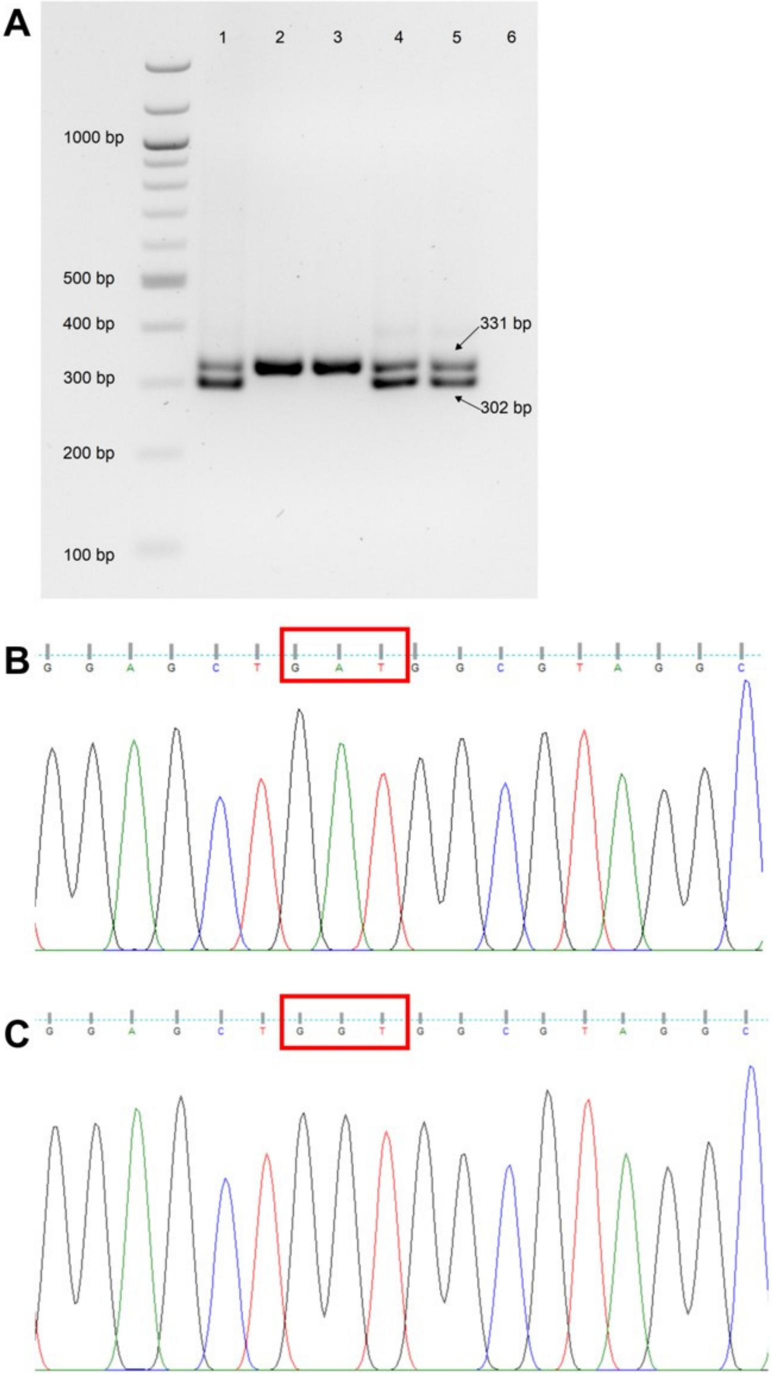

Figure S1. Cell line sex determination and *Kras* G12D mutation verification. A: Jarid 1c/d PCR to verify the sex of the 6606PDA cell line. Lane 1: 6606PDA, lane 2 and 3: female C57BL6/J mice, lane 4 and 5: male C57BL6/J mice, lane 6: negative control. B: Verification of the G12D mutation (G to A

substitution) by Sanger sequencing. C: Sequence of the *Kras* gene in a wildtype C57BL6/J mouse at the same position.

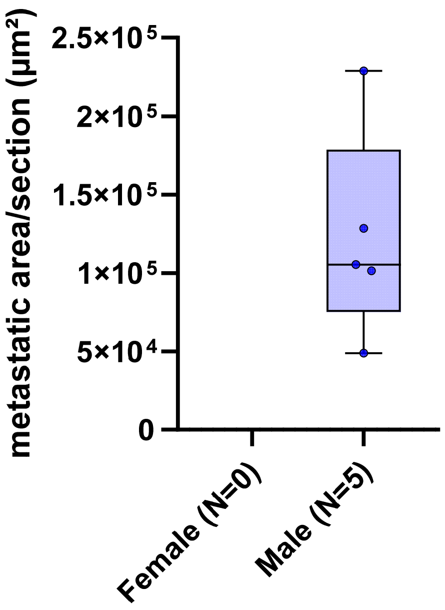

Figure S2. Influence of sex on lung metastasis. Median metastatic area (µm²) in serial histological section of the left lung lobe of female and male mice receiving vehicle solution.

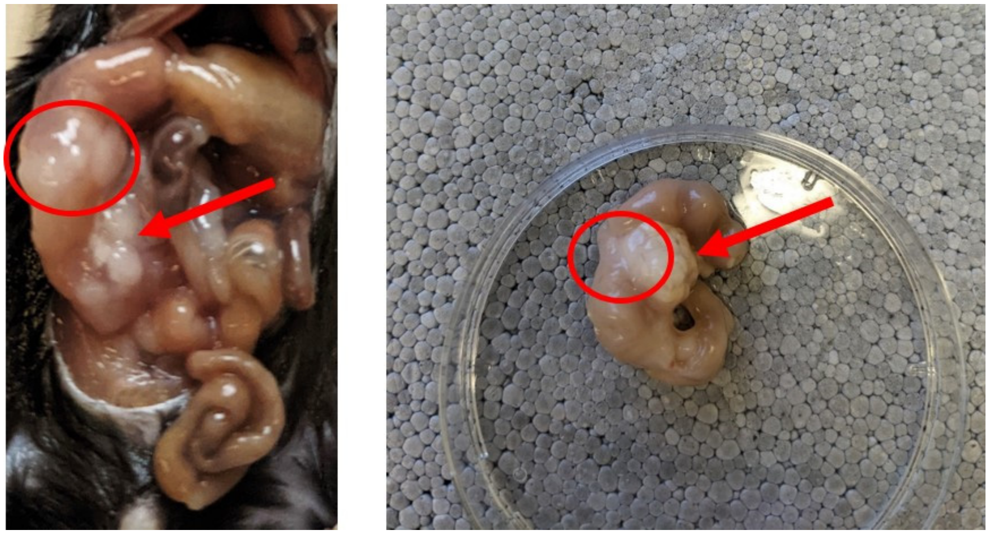

Figure S3. Duodenal invasion of the primary tumor. The red circle shows the invasion into the duodenum and the red arrow shows the primary tumor.

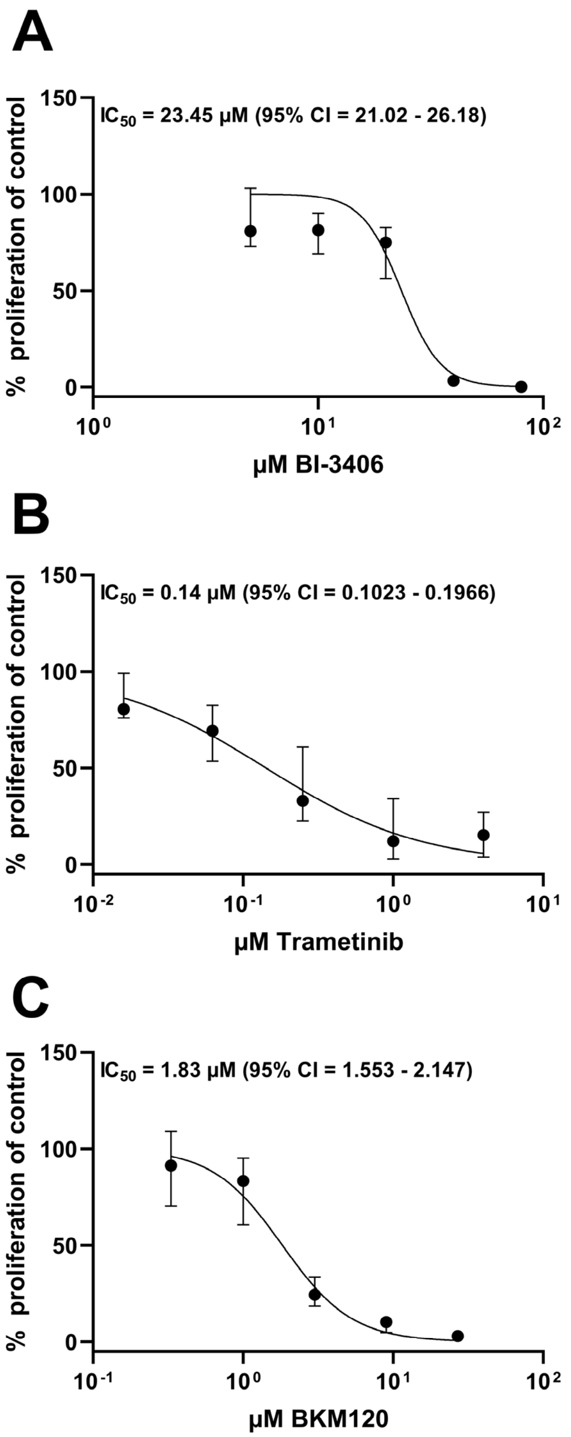

Figure S4. IC_{50} values for inhibition of proliferation (BrdU) for each compound. IC_{50} values for inhibition of proliferation (BrdU) of 6606PDA cells for BI-3406 (A), trametinib (B) and BKM120 (C). Data are shown as median with range. N = 5 for all compounds and concentrations tested.

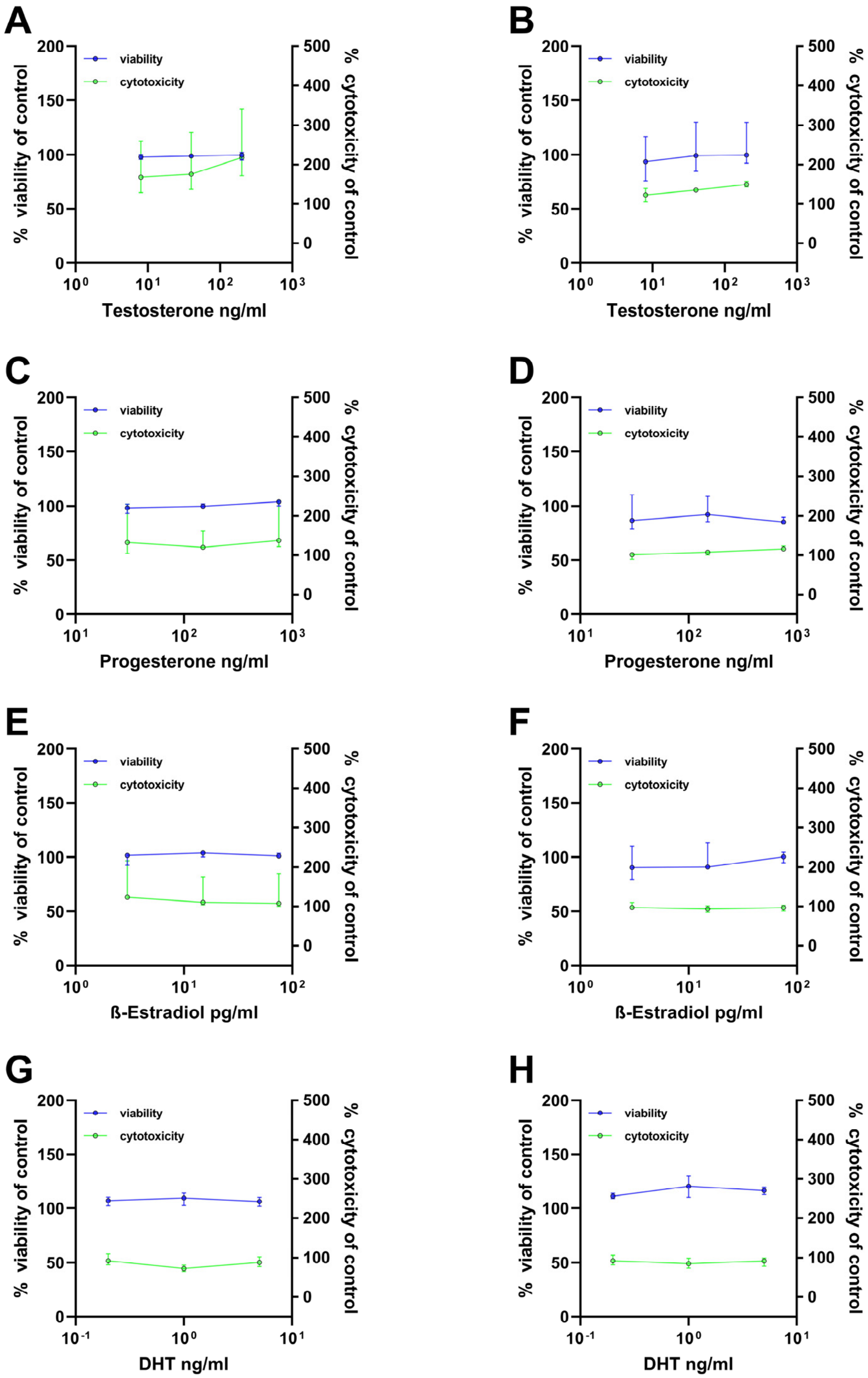

Figure S5. Effects of physiological and supraphysiological sex hormone concentrations on viability and cell death of 6606PDA cells. The effects of physiological and supraphysiological concentrations of testosterone (A, B), progesterone (C, D), 17β–estradiol (E, F), and dihydrotestosterone (G, H) have been tested in cells cultured with either 10% FCS (A, C, E, G) or 2% FCS (B, D, F, H) for 48 h. Data are shown as median with range. N = 3 for all sex hormones and concentrations tested.

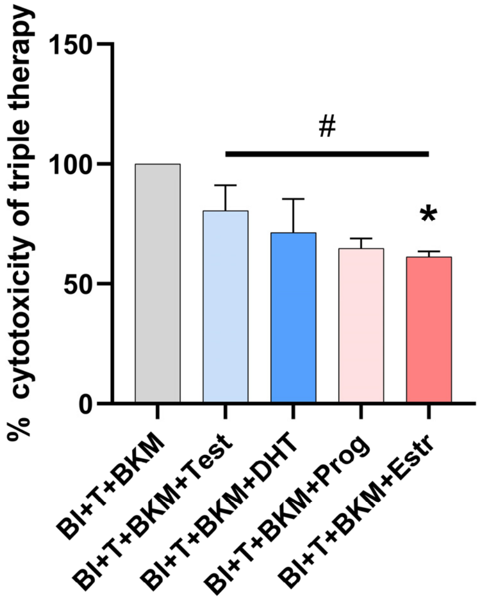

Figure S6. Impact of sex hormones on the efficacy of the combinatorial therapy. The impact of physiological concentrations of either testosterone (8 ng/ml), dihydrotestosterone (0.2 ng/ml), progesterone (30 ng/ml) or 17β-estradiol (3 pg/ml) on the efficacy of the combinatorial therapy have been tested in 6606PDA cells cultured for 48 h with a combination of BI-3406 (BI, 10 μM), trametinib (T, 0.064 μM) and BKM120 (BKM, 1 μM). Data are shown as bar graphs with median and 95% CI. Each combination has been compared to the control (BI+T+BKM, Kruskal-Wallis test with Dunn's post-hoc test, *$p < 0.05$) or each other (Ordinary one-way ANOVA with Tukey post-hoc test, #$p < 0.05$). N = 3 for all each combination. Test: testosterone, DHT: dihydrotestosterone, Prog: progesterone, Estr: 17β–estradiol.

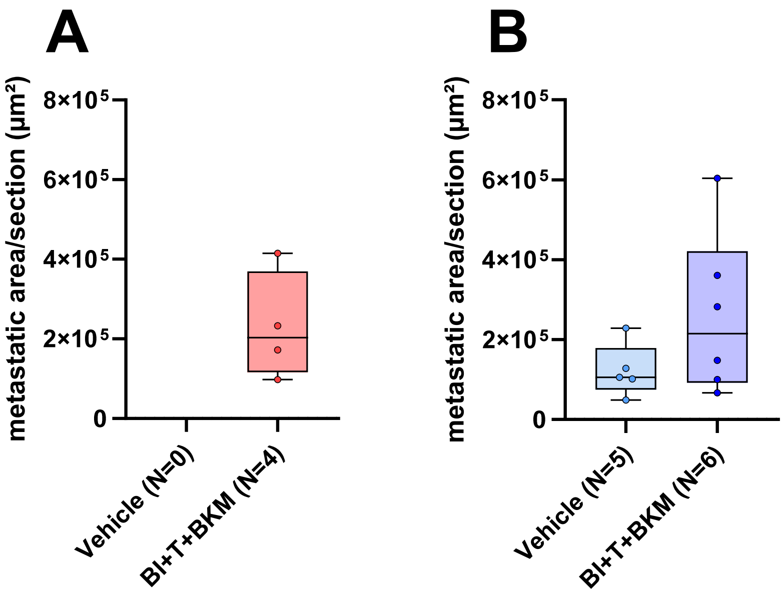

Figure S7. Effect of the combinatorial therapy on the metastatic area in the lung. Median metastatic area (μm²) in serial histological sections of the left lung lobe of female (A) and male (B) mice receiving either vehicle or a combinatorial therapy consisting of BI-3406 (BI), trametinib (T) and BKM120 (BKM).

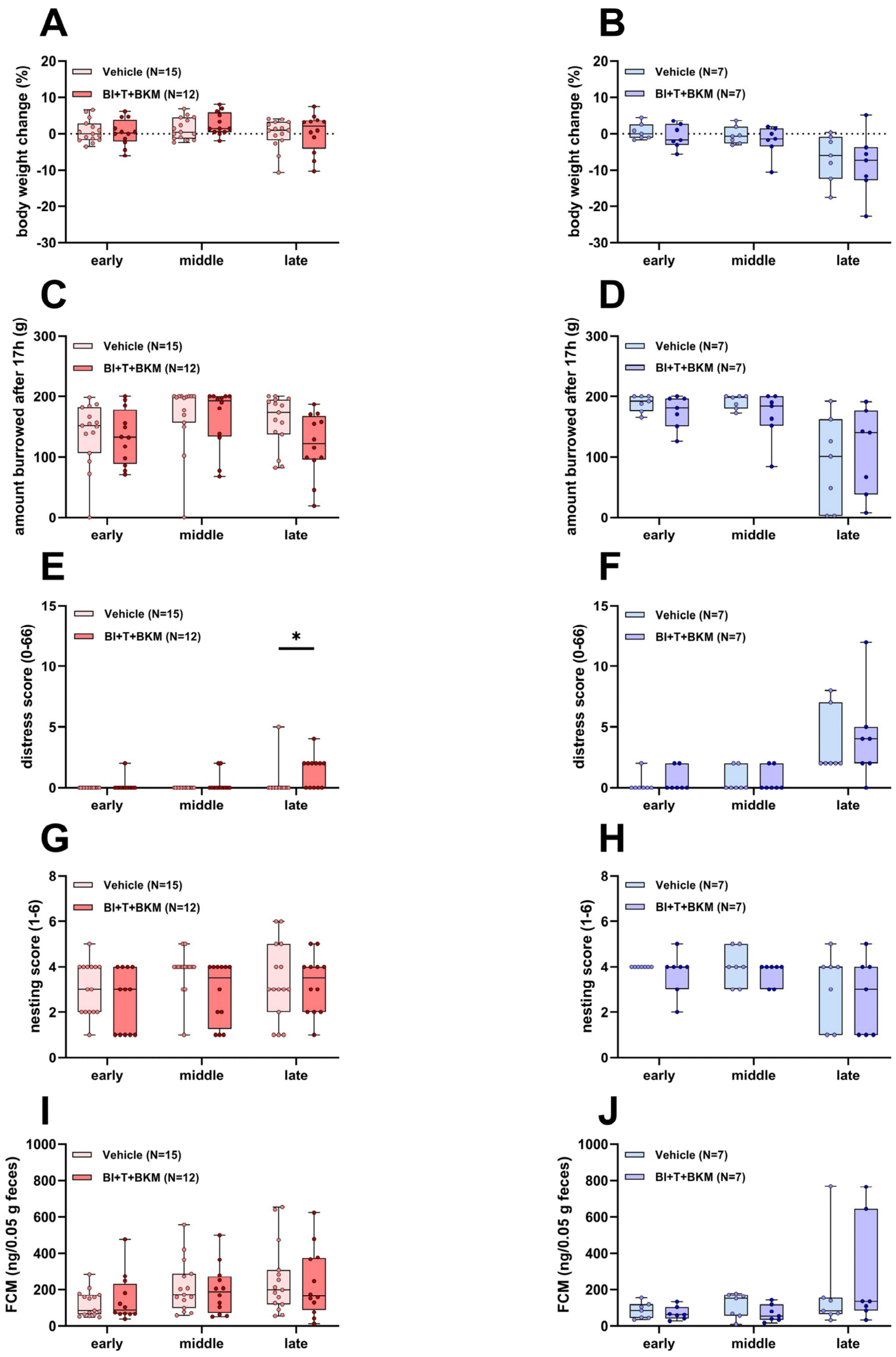

Figure S8. Effect of combinatorial therapy on distress parameters. Body weight (A, B), burrowing activity (C, D), distress score (E, F), nesting activity (G, H), and concentration of fecal corticosterone metabolites (I, J) in surviving female and male mice receiving either vehicle or a combinatorial therapy consisting of BI-3406 (BI), trametinib (T) and BKM120 (BKM) (Two-way repeated measures ANOVA with Sidak's post-hoc test, *p < 0.05). Early, middle and late refer to the experimental phases as defined in the methods section.

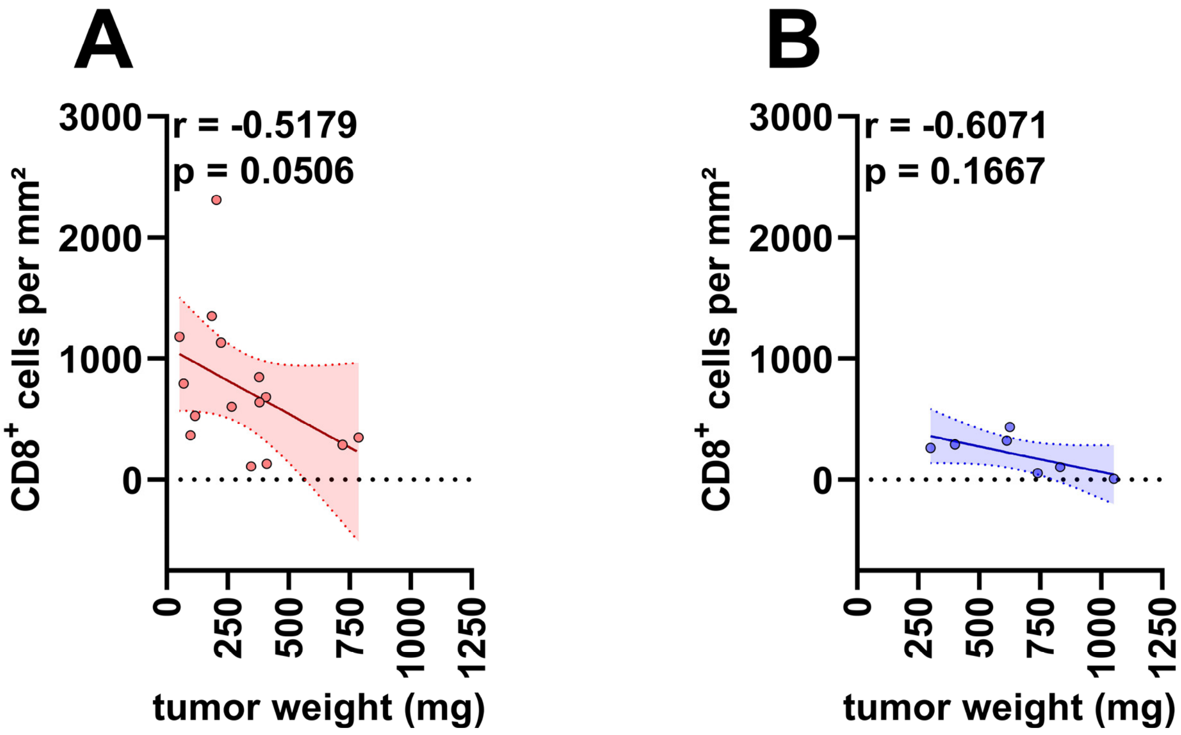

Figure S9. Correlation between the amount of CD8⁺ cells and tumor weight in vehicle-treated animals. Correlation plots (Spearman correlation) with linear regression including confidence intervals (shaded area) of CD8⁺ cells and tumor weight of vehicle-treated female (A, N = 15) and male (B, N = 7) mice.

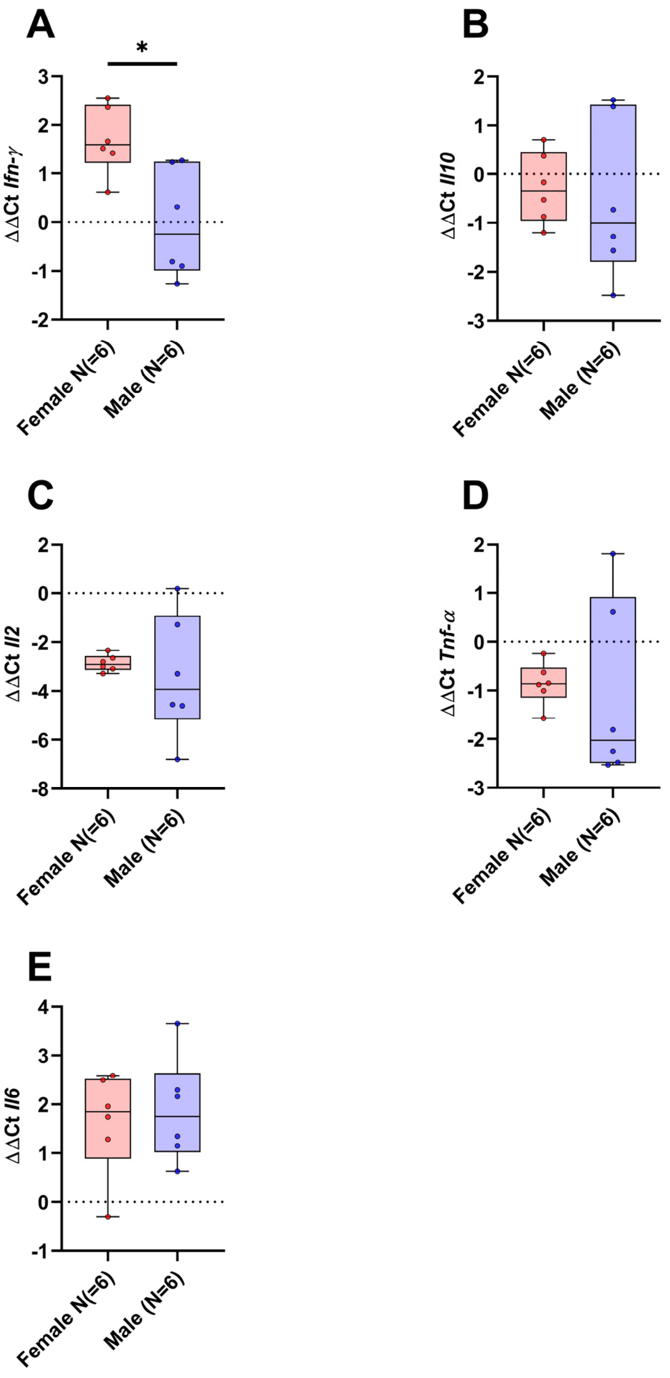

Figure S10. Comparison of relative gene expression of *Ifn-γ*, *Il10*, *Il2*, *Tnf-α* and *Il6* between both sexes. Comparison of relative gene expression of *Ifn-γ* (A, unpaired t-test, $*p < 0.05$), *Il10* (B, unpaired t-test, ns), *Il2* (C, unpaired t-test, ns), *Tnf-α* (D, unpaired t-test, ns). and *Il6* (E, unpaired t-test, ns) between a subset of female and male mice treated with vehicle.

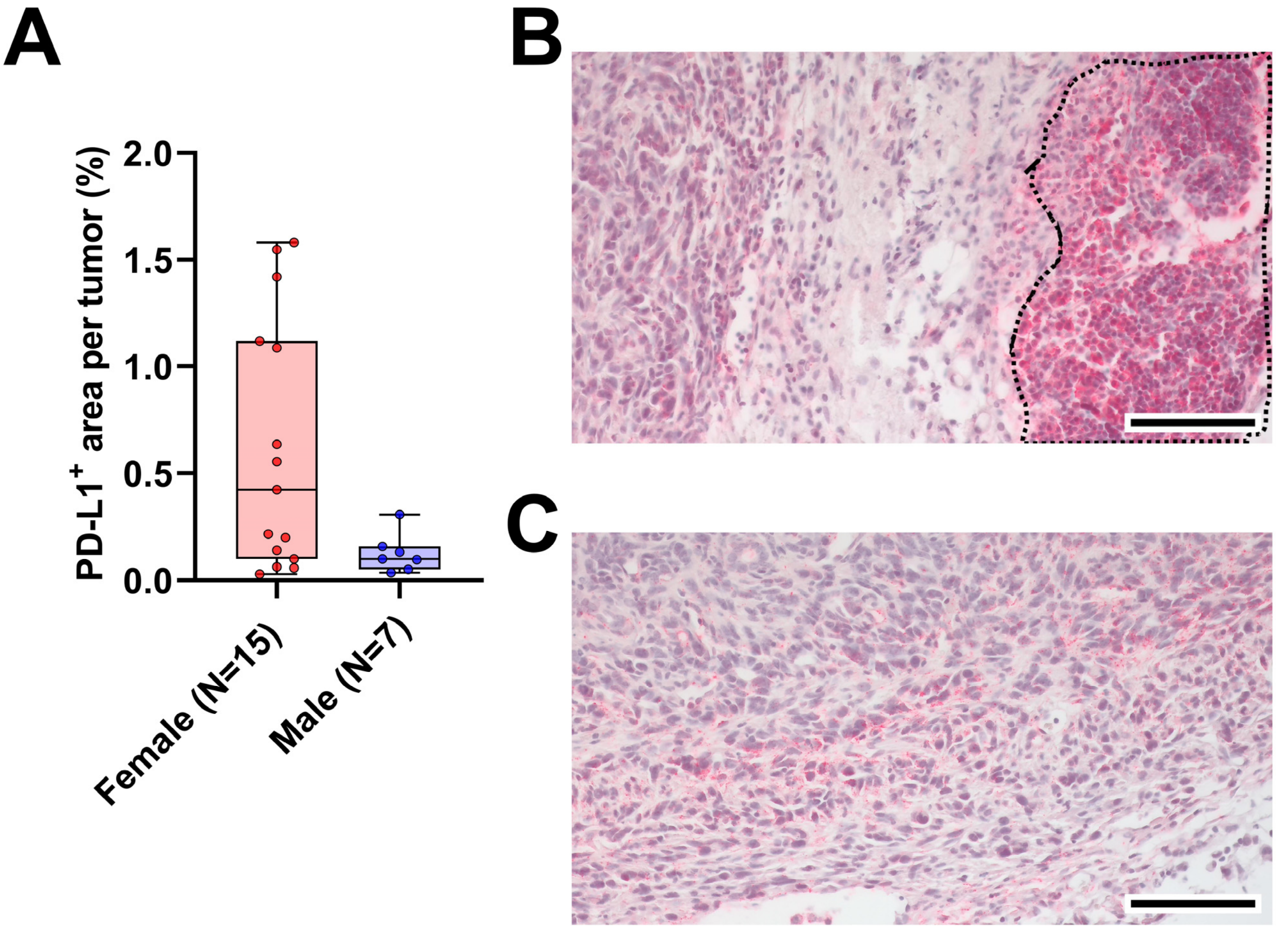

Figure S11. PD-L1 expression in tumors of female and male mice treated with vehicle. A: Comparison of PD-L1-positive area in tumors of vehicle-treated female and male mice (Mann-Whitney test, p = 0.0556). B, C: Representative histological sections of anti-PD-L1 stained tumors of surviving female (B) and male (C) vehicle-treated mice (scale bar = 100 µm). The outlined area in B highlights an inflammatory lesion predominantly seen in female mice.

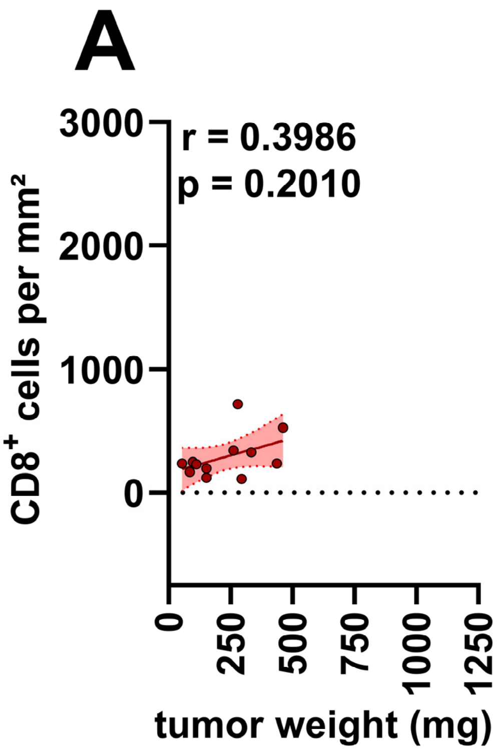

Figure S12. Correlation between the amount of $CD8^+$ cells and tumor weight in animals treated with BI-3406, trametinib and BKM120. Correlation plots (Spearman correlation) with linear regression including confidence intervals (shaded area) of $CD8^+$ cells and tumor weight of drug-treated female (A, N = 12) and male (B, N = 7) mice.

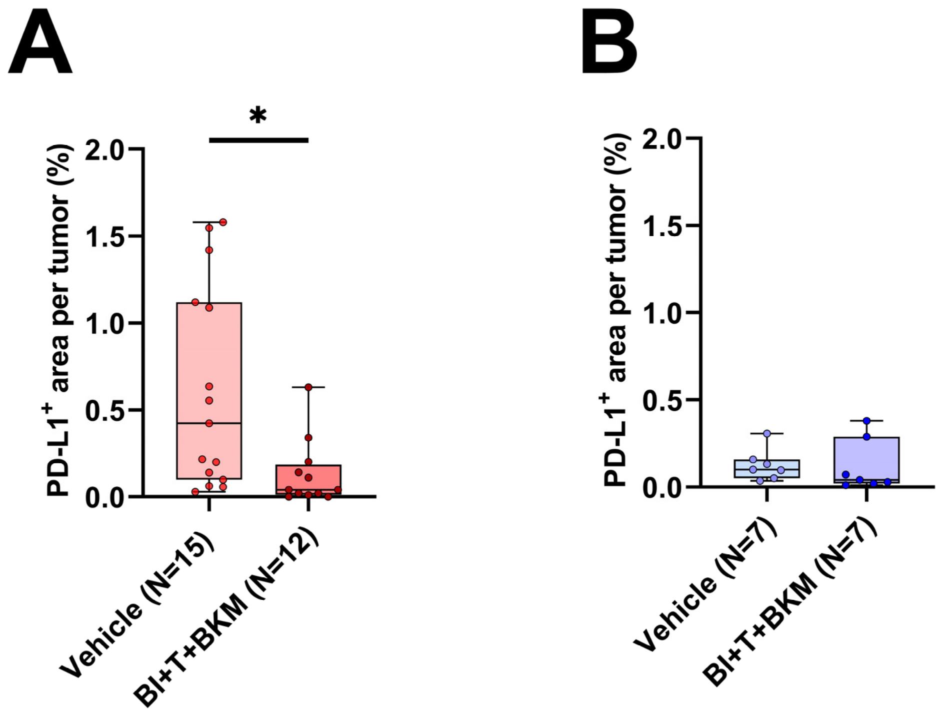

Figure S13. PD-L1 expression in tumors of female and male mice treated with vehicle or BI-3406, trametinib and BKM120. Comparison of PD-L1-positive area in tumors of vehicle and drug-treated female (A) and male (B) mice (Mann-Whitney test,* $p < 0.05$).

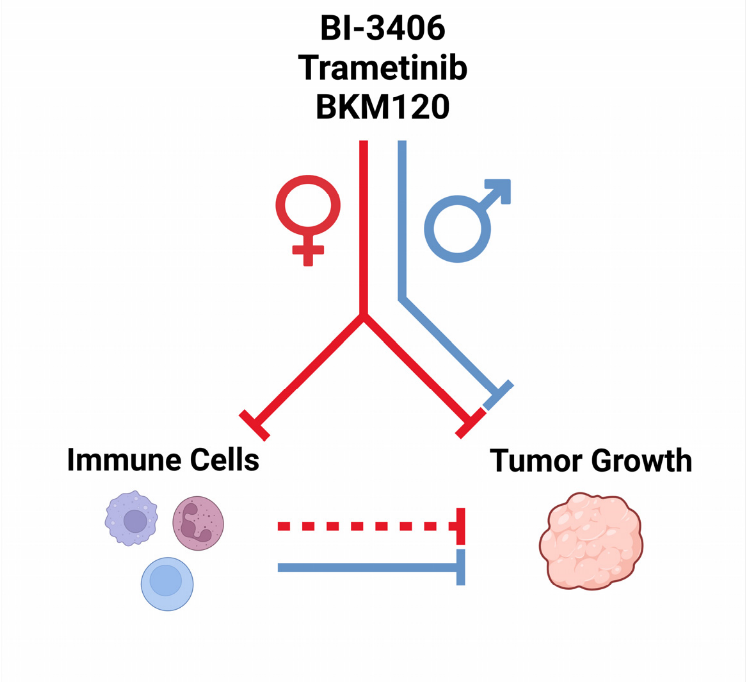

Figure S14. Visualization of hypothesized sex-specific effects. The combination of BI-3406, trametinib and BKM120 reduces tumor growth in both sexes. This reduction is influenced by the inhibition of immune cells predominantly in female animals. Made with biorender.com.

Table S1. Primers used for PCR and sequencing.

**Jarid1c/1d forward**	**CTGAAGCTTTTGGCTTTGAG**
Jarid 1c/1d reverse	CCACTGCCAAATTCTTTGG
Kras-Exon 1-G12D fw2 259-278	TGGTTCCCTAACACCCAGTT
Kras-Exon 1-G12D rv2 625-649	TTAGAGTTTTACACACAAAGGTGAG
Kras-Exon 1-G12D fw1 400-419 (used for sequencing)	TCTTTTTCAAAGCGGCTGGC

Table S2. PCR conditions (Jarid 1c/d).

**Mastermix**	**VWR Taq DNA Mastermix (Avantor, Darm-stadt, Germany)**
Initial denaturing	94°C for 5 min
Denaturing (40x)	94°C for 20 s
Annealing (40x)	54°C for 1 min
Extension (40x)	72°C for 40 s

Table S3. PCR conditions (KRAS G12D and WT).

**Mastermix**	**PowerUp™ SYBR™ Green Master Mix (Ap-plied Biosystems, Waltham, USA)**
UDG activation	50°C for 2 min
Activation (Dual-Lock™ DNA polymer-ase)	95°C for 2 min
Denaturing (40x)	95°C for 15 s
Annealing (40x)	55-60°C for 15 s
Extension (40x)	72°C for 1 min

Table S4. MRI sequences and parameters.

**Parameters**	**axial**	**sagittal**	**coronal**
TE/TR (ms)	25/3476	35/4553	25/2500
Rare factor	8	8	8
Averages	4	4	4
FoV (mm)	320 x 200	32 x 20	32 x 32
Matrix size	255 x 160	240 x 160	255 x 255
Voxel size (mm)	0.125 x 0.125	0.125 x 0.125	0.125 x 0.125
Slice thickness (mm)	0.8	0.8	0.8
Slices	42	40	22
Acquisition time (min:s)	4:38	6:04	5:47

Table S5. Parameters of the triple quadrupole interface.

**Interface ESI parameter**	**Value**
Nebulizing gas flow	3 L/min
Heating gas flow	10 L/min
Interface temperature	300 °C
Desolvation temperature	526 °C
DL temperature	250 °C
Heat block temperature	400 °C
Dry gas flow	10 L/min

Table S6. Mass spectrometric parameters of BKM120, BI-3406, trametinib and the internal standard acridine orange.

Substance	Precursor (*m/z*)	Product (*m/z*)	Dwell time (msec)	Q1 Pre Bias (V)	CE (V)	Q3 Pre Bias (V)	Measuring mode	Measuring time (min)
BKM120	411.3	367.1	515	-10	-35	-26	positive	0-3.5
BKM120	411.3	307.0	515	-11	-40	-22	positive	0-3.5
BKM120	411.3	238.95	515	-11	-46	-26	positive	0-3.5
BI-3406	463.3	205.95	100	-20	-35	-20	positive	4-6.5
BI-3406	463.3	190.9	100	-20	-50	-20	positive	4-6.5
BI-3406	463.3	276.05	100	-19	-28	-21	positive	4-6.5
BI-3406	461.2	375.15	100	13	33	12	negative	4-6.5
BI-3406	461.2	390.2	100	17	24	13	negative	4-6.5
BI-3406	461.2	331.1	100	13	52	14	negative	4-6.5
Acridine Orange	266.3	250.1	100	-14	-35	-28	positive	4-6.5
Acridine Orange	266.3	234.0	100	-14	-52	-26	positive	4-6.5
Acridine Orange	266.3	222.1	100	-14	-34	-24	positive	4-6.5
Trametinib	616.1	490.85	100	-20	-34	-19	positive	6-8
Trametinib	616.1	254.15	100	-20	-40	-19	positive	6-8
Trametinib	616.1	226.05	100	-20	-50	-17	positive	6-8
Trametinib	614.1	531.0	100	24	28	24	negative	6-8
Trametinib	614.1	511.05	100	24	31	24	negative	6-8
Trametinib	614.1	126.9	100	24	50	11	negative	6-8

Table S7. TaqMan qPCR conditions.

Mastermix	TaqMan™ Universal Master Mix II, (Applied Biosystems, Waltham, USA)
UNG incubation	50°C for 2 min
Enzyme activation	95°C for 10 min
Denaturing (40x)	95°C for 15 s
Anneal / Extend (40x)	60°C for 1 min

Table S8. Measured concentrations (LC-MS/MS) of therapeutics in plasma, liver and kidney (median with 5 – 95% confidence interval).

Sex	Sample	BI-3406	Trametinib	BKM120
Male	Plasma (N = 4)	2.1 µM (1.2 – 4.6)#	-*	2.6 µM (1.8 – 6.0)
	Liver (N = 3)	3.9 ng/mg (2.5 – 5.4)	0.42 ng/mg (0.089 – 0.69)	3.0 ng/mg (1.3 – 5.1)
	Kidney (N = 3)	2.2 ng/mg (2.0 – 3.0)	0.13 ng/mg (0.084 – 0.17)	0.48 ng/mg (0.33 – 2.5)
Female	Plasma (N = 6)	2.7 µM (0.82 – 4.2)	-*	4.6 µM (2.4 – 6.4)
	Liver (N = 3)	5.1 ng/mg (0.45 – 10)	0.39 ng/mg (0.30 – 0.48)§	2.4 ng/mg (0.31 – 8.8)
	Kidney (N = 3)	2.7 ng/mg (0.055 – 9.1)	0.21 ng/mg (0.19 – 0.24)§	0.47 ng/mg (0.059 – 7.2)

25% of values below limit of detection (LOD). * 100% of values below limit of detection (LOD). § 33% of values below limit of detection (LOD).

Table S9. p-values for Figure 5.

Tested Difference	p-value (Mann-Whitney test)	p-value (unpaired t-test)
AST (female vehicle vs. therapy)	0.6923	-
ALT (female vehicle vs. therapy)	0.4634	-
creatinine (female vehicle vs. therapy)	-	0.0604

LDH (female vehicle vs. therapy)	0.6483	-
c-peptide (female vehicle vs. therapy)	**0.0026**	-
AST (male vehicle vs. therapy)	-	0.6382
ALT (male vehicle vs. therapy)	-	0.7057
creatinine (male vehicle vs. therapy)	-	0.1680
LDH (male vehicle vs. therapy)	-	0.1686
c-peptide (male vehicle vs. therapy)	**0.0111**	-
